# Evaluation of the Development of Post-Vaccination Immunity against Selected Bacterial Diseases in Children of Post-Solid-Organ-Transplant Mothers

**DOI:** 10.3390/vaccines12060565

**Published:** 2024-05-22

**Authors:** Tomasz Ginda, Karol Taradaj, Olga Tronina, Anna Stelmaszczyk-Emmel, Bożena Kociszewska-Najman

**Affiliations:** 1 Department of Neonatology and Rare Diseases, Faculty of Health Sciences, Medical University of Warsaw, 02-091 Warsaw, Poland; tomasz.ginda@wum.edu.pl (T.G.); bnajman@wum.edu.pl (B.K.-N.); 2 Poland Department of Transplantation Medicine, Nephrology and Internal Diseases, Faculty of Medicine, Medical University of Warsaw, 02-091 Warsaw, Poland; 3 Department of Laboratory Diagnostics and Clinical Immunology of Developmental Age, Faculty of Medicine, Medical University of Warsaw, 02-091 Warsaw, Poland

**Keywords:** immunogenicity, immunosuppressive drugs in pregnancy, transplantation, safety of vaccination, anti-bacterial vaccination, children

## Abstract

Pregnancy after organ transplantation is considered high-risk and requires supervision in specialized centers. The impact of immunosuppression on the developing fetus is still the subject of research. It has been shown that it affects lymphocyte populations in the first year of life. For this reason, researchers suggest postponing mandatory infant vaccinations. The aim of the study was to analyze the influence of intrauterine exposure of the fetus to immunosuppression on the immunogenicity of protective vaccinations against selected bacterial pathogens. The ELISA method was used to determine the concentration of post-vaccination IgG antibodies against diphtheria, tetanus, pertussis, tuberculosis, *H. influenzae* type B, and *S. pneumoniae* in 18 children of mothers who underwent organ transplantation. The results were compared with the control group (n = 21). A comparison of the incidence of adverse post-vaccination reactions between the analyzed groups was also performed. There were no statistically significant differences in the immunogenicity of the analyzed vaccines between children of mothers who underwent organ transplantation and the age-matched general pediatric population. There were no differences in the incidence of adverse post-vaccination reactions between the analyzed groups. The obtained results do not indicate the need to modify the current protective vaccination schemes against bacterial pathogens in children of mothers who underwent organ transplantation.

## 1. Introduction

Transplantology is one of the fastest growing fields of medicine. Due to the development of this field, there is a steadily increasing age of survival after transplantation, sometimes comparable to the general population. Thus, the development of peri-transplantation knowledge, focused not so much on maintaining the function of the transplanted organ, but also holistic measures aimed at improving the quality of life, is becoming more important. One of the most important aspects of social life, as well as human physiology, is reproduction and the desire to have offspring. In the past, pregnancy in solid-organ transplant patients was a debatable issue. Initially, the relatively short survival period of patients after transplantation surgery due to the imperfection of surgical techniques, as well as post-transplantation care, meant that pregnancy in organ recipients was not recommended. The first full-term pregnancy of a transplant patient was described in 1963 by Murray et al. [1]. It was a patient after a kidney transplant. It is now known that pregnancy after organ transplantation is possible. Current immunosuppressive regimens in pregnancy allow one to maintain the function of the transplanted organ and to carry to term. However, each pregnancy in an post-organ-transplant patient is considered a high-risk pregnancy. Patients should be monitored by a multidisciplinary team with relevant experience in managing pregnancies of post-transplant patients, preferably in higher-level referral facilities. Pregnancy in post-organ-transplant patients has been shown to have an increased risk of maternal–fetal complications. Despite the available knowledge and experience of clinicians, pregnancy after organ transplantation is associated with a 10-fold lower probability of giving a birth to a live baby than that in the general population [2]. An increased risk of hypertension, preeclampsia, and eclampsia has been observed in pregnant post-transplant women. Fetal complications include growth restriction, preterm delivery, and increased incidence of cesarean section [3,4,5,6,7,8]. Knowledge of the long-term effects of intrauterine fetal exposure to immunosuppressive drugs is incomplete. Few papers are available in the literature assessing, among other things, the development of children of post-transplant mothers in the context of selected biochemical parameters [9], lipid metabolism [10], immune system development [11], neurological system [12], birth parameters, and the course of pregnancy and delivery [3,4,5,6,7,8]. Intrauterine fetal exposure to immunosuppressive drugs has an impact on the development of the child’s immune system. It has been shown that during the first year of life, children of post-transplant mothers show a reduced number of T and B lymphocytes compared to the general population [13,14,15]. For this reason, it seems to be important to assess whether children from this population also show a different post-vaccination response to the general pediatric population. Vaccination is an important aspect for the proper development of a child, preventing life-threatening infectious diseases. Therefore, knowing whether vaccination schedules in this group of children should be implemented the same or differently from the common immunization calendar is essential. In the literature so far, only a few papers have been published comparing the immunogenicity of vaccination in children of organ recipient mothers which concerned the response to vaccination against selected viral and bacterial diseases [16,17]. The conclusions of the papers were based on studies conducted on small groups of patients, and each time the authors stressed the need for expanded studies on larger, more representative groups of patients. Significant pathogens in both childhood and adulthood include pathogens against which there are immunizations. Assessing the impact of intrauterine fetal exposure on the long-term response to vaccination directed against bacterial diseases has not yet been analyzed by researchers. In view of the fact that this issue appears to have significant clinical relevance it has become the subject of this paper.

The aim of the study was to evaluate the immunogenicity of immunization against diphtheria, tetanus, pertussis, tuberculosis, *Hemophilus influenzae* type b, and *Streptococcus pneumoniae* in children of mothers after liver or kidney transplantation and its comparison to children from the general population. An additional aim was to assess the incidence of adverse reactions after vaccination in the groups analyzed.

## 2. Material and Methods

The study was conducted in 2021–2022. The study included two groups of children between the ages of 6 and 16 born between 2008 and 2014. The study group consisted of 18 children of post-solid-organ-transplant mothers: 9 children of liver recipient mothers and 9 children of kidney recipient mothers. The children’s mothers took immunosuppressive drugs during pregnancy in accordance with current standards of post-transplant care. The control group consisted of 21 healthy, gender-matched children from the general pediatric population. All children were vaccinated in accordance with the immunization calendar in effect in Poland. In order to ensure the greatest homogeneity of the groups in terms of the number of immunization doses received, children between the ages of 6 and 16 were selected, since vaccination against bacterial diseases, the subject of this study, is not performed during this age range. All children received 1 dose of BCG (Bacillus Calmette–Guérin) tuberculosis vaccine, 4 doses of DTP (Diphtheria, Tetanus, Pertussis), 4 doses of Hib (*Hemophilus influenzae* type b) vaccine, and 3 doses of 13-valent pneumococcal vaccine (PCV-13)

Detailed characteristics of the groups, including the inclusion and exclusion criteria, as well as the regimens of immunosuppressive treatment used are summarized in Table 1 and Table 2.

Data on the occurrence of adverse events related to vaccinations were collected from analyses of patients’ medical records as well as on the basis of questionnaires completed by parents regarding the medical history of children. The severity of the local and systemic AEFIs was graded on a scale of 1 to 4 based on the guidelines of the U.S. Food and Drug Administration [18].

The study was conducted in accordance with the principles of the Declaration of Helsinki, and the study protocol was approved by the Medical University of Warsaw Bioethics Committee (Approval no. KB/161/2021).

A sample of 3 mL of venous blood was taken from each child into a clot activator tube. The material was then centrifuged (10,000 rpm, 5 min). The separated blood plasma was frozen at −80 degrees C. After obtaining a set of samples, the material was thawed and analyzed by ELISA. The following antibodies were analyzed: anti-tuberculosis BCG IgG, anti-*Hemophilus influenzae* B IgG, anti-*S. pneumococcal* vaccine IgG, anti-Tetanus Toxoid IgG. Standardized reagent kits from Alpha Diagnostic Intel were used for ELISA testing. Each sample was tested twice. The tests were performed according to the manufacturer’s instructions. Absorbance was read using a UVM340 plate reader (ASYS, Biogenet, Santa Clara, CA, USA). The results were then analyzed using MikroWin2000 v4 software (Mikrotek La-borsysteme GmbH, Biogenet, Overath, Germany). Absorbance results were converted to antibody concentrations in units (U/mL) as defined by the manufacturer. An average was calculated from the two measurements for each sample, which was used for further statistical analysis. Details are shown in Table 3.

Statistical analysis was performed using StatSoft Statistica 13.1 software. A verification of assumptions for the use of parametric tests was carried out. The normality of the distributions was tested using the Shapiro–Wilk test. Homogeneity of variance was assessed using the Leaven test. In each case, the distributions analyzed did not meet the criteria for parametric tests. In view of the above, a proper statistical analysis was carried out using non-parametric tests. The strongest equivalent of one-way analysis of variance, the Kruskal–Wallis test, was used. Relative to the correlations for which statistically significant differences were shown, a post hoc analysis was performed using the Dunn test with Bonferroni correction. The test was chosen because of the small size of the control and study groups, which is due to the specific nature of the patient group, which includes children of post-transplant mothers.

The median concentrations of immune antibodies were analyzed by the Kruskal–Wallis test, followed by post hoc analysis, separately for each type, i.e., anti-tuberculosis BCG IgG, anti-*Hemophilus influenzae* B IgG, anti-*S. pneumococcal* vaccine IgG, anti-Tetanus Toxoid IgG between children of mothers after organ transplant (liver and kidney combined–LTR + KTR), kidney and liver (subgroups, respectively: KTR and LTR) and children from the control group (no intrauterine exposure to immunosuppressive drugs—Control). A significance level of α = 0.05 was adopted, below which the results were statistically significant. The results of the analyses are summarized in Table 4, Table 5, Table 6 and Table 7 and discussed in Section 3.

## 3. Results

Table 3 shows the detailed results of concentrations for each type of immune antibody.

Table 4, Table 5, Table 6 and Table 7 show the results of the analysis of Dunn’s post hoc test with Bonferroni correction for each type of antibody. The tables do not include the results of analyses for C. Diphteriae and B. Pertusis for which no statistical relationships were shown.

**Figure 1:**
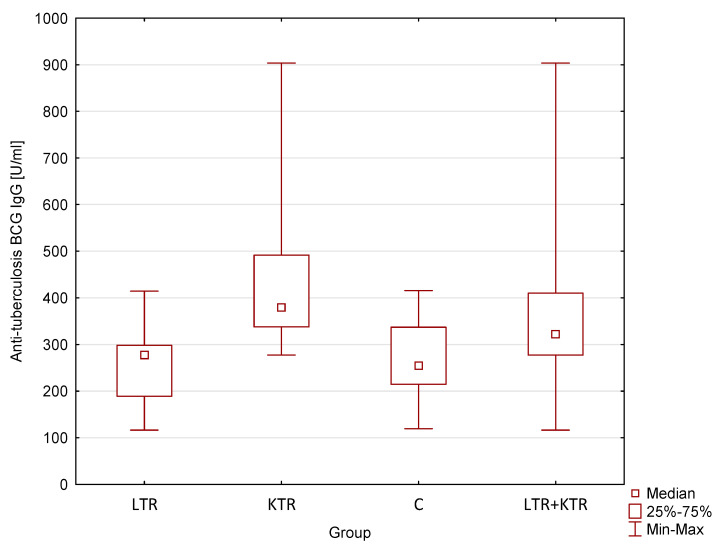
Medians of anti-tuberculosis BCG IgG antibody concentrations.

Table 4 shows the results of post hoc analysis of anti-tuberculosis BCG IgG antibodies. According to the analysis, the median concentration of anti-tuberculosis BCG IgG antibodies in children of post-organ-transplant mothers (both children of liver or kidney recipients and regardless of the type of transplanted organ) was higher than in the control group, in which it amounted to 254.77 U/mL (IQR = 122.32 U/mL). However, only in children of renal transplant mothers (379.15 U/mL; IQR = 154.18 U/mL) statistically significant differences (*p* = 0.03 < α) were shown. In the other subgroups, despite finding a higher median value, the results were not statistically significant (*p* > 0.05). The results are illustrated in Figure 1 in the form of “box-and-whisker” diagrams.

Table 5, Table 6 and Table 7 show the results of post hoc analyses, respectively, for anti-*Hemophilus influenzae* B IgG, anti-*S. pneumococcal* vaccine IgG, *anti-Tetanus* Toxoid IgG. On the basis of Dunn’s post hoc analysis with Bonferroni correction for each of the analyzed antibody types, there were no statistically significant differences between the analyzed subgroups (*p* > 0.05).

A comparative analysis of the incidence of adverse vaccine reactions was also conducted. The incidence of serious and severe vaccine reactions was not observed in the study populations. Among the most common were malaise and redness at the injection site. The incidence of mild vaccine reactions was analyzed using the Mann–Whitney U-test. No differences in their incidence were observed between the study group (KTR + LTR) and the control group were observed (22% vs. 28% *p* > 0.05).

## 4. Discussion

The analysis showed no statistically significant differences in post-vaccination antibodies to diphtheria, tetanus, pertussis, tuberculosis, and pneumococcus between children of liver and kidney recipient mothers and those of the general population. Chronic maternal immunosuppression also applied during pregnancy is not indifferent to the developing fetal immune system. As shown in numerous publications, children of post-solid-organ-transplant mothers in the first 12 months of life have reduced numbers of T lymphocytes (CD3+, CD4+, CD8+) and B lymphocytes (CD19+) compared to children not exposed to immunosuppression during fetal life [13,14,15]. After the first year of life, the white blood cell smear normalizes and no longer shows differences from the general population.

The abnormalities found in the development of the immune system in the first months of life may suggest a poorer response to vaccination in children of post-transplant mothers. The analysis of immunization immunogenicity is a clinically important aspect of interdisciplinary care for this special group of patients.

The topic of the health of children of post-transplant mothers has been covered quite extensively in the works of other researchers. However, the body of work evaluating the immunogenicity and safety of immunization in this patient group available in medical publication databases is limited. For this reason, each newly published work analyzing this issue represents a significant development of the current state of knowledge.

Dinelli et al. [17] conducted a study on a group of 24 children of kidney post-transplant mothers at 7–8 months of age. They showed that median concentrations of antibodies to tetanus, *H. influenzae* type B, and pneumococcus were comparable to the general population (*p* > 0.05). There were also no differences in the incidence of vaccine side effects between the groups. The results were therefore comparable to those obtained by our team.

Baarsma et al. [19] described a clinical case of the child of a post-liver-transplant mother. Antibody concentrations against diphtheria, tetanus, and poliomyelitis in the second year of life were analyzed. Again, the post-vaccination response was adequate.

In our study, we found higher median anti-tuberculosis BCG IgG antibody concentrations in the children of kidney recipient mothers compared to children from the general population (*p* < 0.05). Given the lack of differences for all other vaccine antibodies, the reason for the relationship obtained is unknown. Based on current medical knowledge, it is difficult to explain the reason for the obtained correlation and this needs to be verified in further studies.

As is known, there are many other factors that influence the development of the newborn’s immune system. One of them is breastfeeding and the moment of introducing complementary foods [20]. Breastfeeding, especially in the first period of life, has a positive impact on the development of the newborn’s immunity [20,21,22,23]. The analysis of the impact of these factors was not the subject of our study.

The literature contains the results of few studies assessing the concentration of immunosuppressive drugs used in post-transplantation care in human milk [24,25,26,27,28,29,30]. The results of the analyzed studies clearly indicated that the concentration of cyclosporine in colostrum and mature milk is significantly below the therapeutic level (approx. 1% of the maintenance dose for adults), and the bioavailability after oral ingestion is approximately 28% [24]. Despite the low level of transmission of cyclosporine and its metabolites into breast milk, the benefits of breastfeeding by mothers—organ recipients—is debatable. Clinicians involved in post-transplantation care inform women about the possible positive and negative effects of breastfeeding while receiving immunosuppressive therapy. The choice is left to the mother’s discretion. Perhaps future studies conducted on larger groups of patients will contribute to changing the current recommendations.

The current state of knowledge on the immunogenicity of immunization against bacterial diseases in children of post-transplant mothers is incomplete. The few studies that have been conducted on this issue cannot be the basis for formulating separate guidelines for the vaccination of children of post-organ-transplant mothers. Currently, vaccination schedules for children of recipient mothers are implemented according to the vaccination calendar for children in the general population. The results of the conducted work, as well as the available literature data, do not indicate the need to modify this procedure. Given our results, it seems reasonable to maintain a standard vaccination protocol against bacterial diseases in the group of children of post-transplant mothers, the same as in the general population.

## 5. Conclusions

1. No difference in the immunogenicity of immunization against selected childhood infectious diseases of bacterial etiology (diphtheria, tetanus, pertussis, tuberculosis, pneumococcus) has been found in children of post-solid-organ-transplant mothers compared to the general pediatric population.
2. Increased immunogenicity of the BCG (Bacillus Calmette–Guérin) vaccine has been demonstrated in children of kidney-recipient mothers compared to children of the general population. However, the reason for this relationship is unclear and requires further research on larger patient populations.
3. The safety profile of the bacterial vaccinations analyzed in children of post-solid-organ-transplant mothers is comparable to that in the general pediatric population.
4. Based on the analysis, there is no evidence for the validity of modifying childhood bacterial vaccination schedules in children of post-transplant mothers.
5. It is suggested that existing vaccinations schedules be maintained and that children of organ recipient mothers be vaccinated in accordance with the immunization calendar in effect for children in the general population.
6. Due to the limited number of patients and the small number of publications available in the literature, it is suggested to continue studies on a larger population based on multicenter studies.

## 6. Limitations of the Study

This study analyzes post-vaccination antibody concentrations against diphtheria, tetanus, pertussis, tuberculosis, *Hemophilus influenzae* type b, and *Streptococcus pneumoniae* in children of post-liver/kidney-transplant mothers and in a control group aged 6 to 16 years. The study does not evaluate the post-vaccination response in younger children (under 6 years of age) or older children and adults (over 16 years of age). The test evaluates only the humoral response. The methodology adopted in the study, which relies on the evaluation of post-vaccination antibodies, does not allow for the determination of the cellular response in children of post-transplant mothers. The vaccination rate for the infectious diseases studied in the European Union is high; thus, there is high herd immunity in the area of patient residence and migration. For this reason, it is not possible to infer the effectiveness of immunization (determined as the effectiveness of protection against the occurrence of an infectious disease) only by assessing immunogenicity, both in the population of children of post-transplant mothers and children of the general population from Poland.

In our study a small number of patients were examined (research group—18; control group—21). This results in suboptimal power of the study. The rare group of patients, such as children of mothers after organ transplantation, makes it difficult to conduct research on a larger group of patients. Considering the fact that no other research on this topic, conducted on a larger population, has been published so far, each new study expands the available knowledge about the health of children of mothers after organ transplantation. No statistical analysis of the effect of individual immunosuppressive drugs used by mothers during pregnancy on the immunogenicity of their children’s immunizations was performed in this study. Six independent immunosuppression regimens (cyclosporine + azathioprine + corticosteroid, tacrolimus + azathioprine + corticosteroid, tacrolimus + corticosteroid, tacrolimus in monotherapy, azathioprine + corticosteroid, tacrolimus + azathioprine) were used in the analyzed groups of female liver and kidney recipients. Conducting an analysis of differences would require dividing the 18 patients into six subgroups, and such small groups cannot be the basis for statistical inference. For such an analysis to be carried out, a much larger group of patients would need to be included in the study. To this end, it would be beneficial to carry out a multicenter study conducted in collaboration of centers dealing with the health of children of organ recipient mothers.

## Figures and Tables

**Table 1:** Inclusion and exclusion criteria.

Inclusion Criteria	Exclusion Criteria
Age: 6–16. Maternal immunosuppression during pregnancy due to organ transplantation. Non-use of long-term pharmacotherapy. Immunization in accordance with the Protective Immunization Program applicable in Poland for this year of birth. Informed consent to participate in the study.	Active infection of the respiratory tract, digestive system, and urinary tract within 30 days prior to sample collection (existence of symptoms such as runny nose, cough, body temperature above 38 degrees Celsius, acute diarrhea). Chronic diseases of the digestive system (e.g., Crohn’s disease), respiratory system (e.g., cystic fibrosis), in particular autoimmune diseases, systemic connective tissue diseases, congenital and acquired immunodeficiencies, cancer.

**Table 2:** Detailed description of the study and control groups.

Parameters	Transplant n = 18	Control n = 21	*p* Value (Mann–Whitney U Test)
**Children**			
Male	7 (+39%)	9 (+43%)	*p* > 0.05
Female	11 (61%)	12 (57%)	*p* > 0.05
Mean ± SD age	12.11 ± 3.16	9.05 ± 3.07	*p* > 0.05
Chronic diseases	2 (11%)	3 (14%)	*p* > 0.05
History of hospitalization	0 (0%)	1 (5%)	*p* > 0.05
History of vaccination AEs			
Mild (grade 1)	4 (+22%)	6 (+28%)	*p* > 0.05
Moderate (grade 2)	0 (0%)	0 (0%)	*p* > 0.05
Severe (grade 3)	0 (0%)	0 (0%)	*p* > 0.05
Potentially life threatening (grade 4)	0 (0%)	0 (0%)	*p* > 0.05
**Type of Tx**			
Kidney	9		
Liver	9		
**Immunosuppressive schemes during pregnancy**	**Children of KTRs**	**Children of LTRs**	
Cyclosporine + azathioprine + steroid	6 (+67%)	0	
Tacrolimus + azathioprine + steroid	3 (+33%)	3 (+33%)	
Tacrolimus + steroid	0	1 (+11%)	
Tacrolimus	0	2 (+22%)	
Azathioprine + steroid	0	1 (+11%)	
Tacrolimus + azathioprine	0	2 (+22%)	

**Table 3:** Medians with quartile ranges and means with standard deviations of immune antibody concentrations in the children in study and control groups.

	Study Group						Control Group	
	Children of Mothers Who underwent Organ Transplantation in Total (LTR + KTR)		Children of Mothers Who underwent Kidney Transplantation (KTR)		Children of Mothers Who underwent Liver Transplantation (LTR)		Children from the Control Group	
	Median Concentration of Antibodies IgG (IQR) [U/mL]	Mean Concentration of Antibodies IgG +/− SD [U/mL]	Median Concentration of Antibodies IgG (IQR) [U/mL]	Mean Concentration of Antibodies IgG +/− SD [U/mL]	Median Concentration of Antibodies IgG (IQR) [U/mL]	Mean Concentration of Antibodies IgG +/− SD [U/mL]	Median Concentration of Antibodies IgG (IQR) [U/mL]	Mean Concentration of Antibodies IgG +/− SD [U/mL]
*C. diphteriae*	24.6 (39.85)	40.07 +/− 37.30	26.63 (39.34)	39.19 +/− 29.33	19.07 (21.64)	40.94 +/− 45.76	20.92 (32.22)	35.33 +/− 33.28
*B. pertusis*	2580.24 (10800.29)	46,769.97 +/− 84,326.64	3983.48 (9566.65)	47,827.15 +/− 86,349.95	2110.48 (1589.01)	45,712.79 +/− 87,475.77	13,490.10 (198,515.70)	93,800.13 +/− 105,233.80
*M. tuberculosis*	321.85 (132.60)	350.37 +/− 175.22	379.15 (154.18)	443.51 +/− 193.07	277.90 (109.47)	257.23 +/− 91.94	254.77 (122.32)	273.67 +/− 80.88
*H. influenzae*	546.10 (1848.57)	839.57 +/− 685.17	678.33 (1761.52)	1022.39 +/− 672.41	332.39 (1775.40)	656.75 +/− 685.76	890.50 (1838.62)	951.82 +/− 612.24
*N. meningitidis*	0 (395.15)	261.37 +/− 435.98	143.05 (706.38)	409.64 +/− 548.63	0 (0.82)	113.09 +/− 231.22	0 (339.45)	203.29 +/− 300.65
*S. pneumoniae*	1254.26 (54.93)	1144.69 +/− 287.02	1267.05 (18.00)	1262.42 +/− 33.84	1231.06 (386.36)	1026.95 +/− 377.79	1259.47 (30.30)	1133.42 +/− 302.02
*C. tetani*	80.76 (94.98)	123.05 +/− 119.32	140.81 (217.10)	183.49 +/− 139.12	53.52 (82.02)	62.61 +/− 51.77	49.60 (132.80)	83.31 +/− 79.12

**Table 4:** Results of statistical analysis of Anti-tuberculosis BCG IgG antibody concentrations.

*Anti-tuberculosis* BCG IgG—the Kruskal–Wallis *p*-Value Test				
	LTR	KTR	Control	LTR + KTR
LTR		0.05	1.00	0.79
KTR	0.05		0.03	0.79
Control	1.00	0.03		0.72
LTR + KTR	0.79	0.79	0.72	

**Table 5:** Results of statistical analysis of anti-*Hemophilus influenzae* B IgG antibody concentrations.

Anti-*Hemophilus influenzae* B IgG—the Kruskal–Wallis *p*-Value Test				
	LTR	KTR	Control	LTR + KTR
LTR		0.26	0.28	1.00
KTR	0.26		1.00	1.00
Control	0.28	1.00		1.00
LTR + KTR	1.00	1.00	1.00	

**Table 6:** Results of statistical analysis of anti-*S. pneumococcal* vaccine IgG antibody concentrations.

Anti-*S. pneumococcal* Vaccine IgG—the Kruskal–Wallis *p*-Value Test				
	LTR	KTR	Control	LTR + KTR
LTR		0.43	1.00	1.00
KTR	0.43		1.00	1.00
Control	1.00	1.00		1.00
LTR + KTR	1.00	1.00	1.00	

**Table 7:** Results of statistical analysis of anti-Tetanus Toxoid IgG antibody concentrations.

Anti-Tetanus Toxoid IgG—The Kruskal–Wallis *p*-Value Test				
	LTR	KTR	Control	LTR + KTR
LTR		0.16	1.00	1.00
KTR	0.16		0.17	1.00
Control	1.00	0.17		1.00
LTR + KTR	1.00	1.00	1.00	

## Data Availability

The original contributions presented in the study are included in the article, further inquiries can be directed to the corresponding author.
